# Patients’ Self-Reported Disability Weights of Top-Ranking Diseases in Thailand: Do They Differ by Socio-Demographic and Illness Characteristics?

**DOI:** 10.3390/ijerph17051595

**Published:** 2020-03-02

**Authors:** Jiraluck Nontarak, Sawitri Assanangkornchai, Sarah Callinan

**Affiliations:** 1 Health Systems Research Institute, Nonthaburi 11000, Thailand; 2 Epidemiology Unit, Faculty of Medicine, Prince of Songkla University, Songkhla 90110, Thailand; 3 Centre for Alcohol Policy Research, Latrobe University, Victoria 3086, Australia

**Keywords:** disability weights, major depressive disorder, alcohol use disorder, osteoarthritis, valuation method

## Abstract

Little is known about the impact of methodological decisions on estimating disability weights among patients with mental and physical disorders, and the effects of socio-demographic status on the estimation of these weights. A cross-sectional study was conducted in three hospitals in southern Thailand to describe the disability weights based on different valuation methods. Altogether, 150 patients with major depressive disorder, 150 with alcohol use disorder, and 150 with osteoarthritis with varying levels of severity were recruited. Using a face-to-face interview, all patients completed a visual analogue scale (VAS) and were randomly assigned to complete either the European Quality of Life-5 Dimensions (EQ-5D) or Time-trade-off (TTO) instrument to estimate their current utility score, which was consequently transformed to a disability weight. Significant differences were found between disability weights derived from the three instruments for the same disease, with the VAS providing the highest and EQ-5D the lowest weights. Patients with major depressive disorder had the highest disability weight while those with osteoarthritis had lowest by most methods. Patients’ socio-demographics do not affect how they perceive their disability or health condition. Our findings highlight the importance of carefully selecting methods of valuing disability weights, which can rely on disease specific conditions.

## 1. Introduction

Globally, major depressive disorders (MDD) were in the top three leading causes for non-fatal health loss, accounting for 32.8 million years lived with disability (YLD) in 2017 (4.4% of YLD) and increased steadily by 14.3% from 2007 to 2017 [1]. Osteoarthritis is the leading cause of disability in the elderly. Between 2006 and 2016, hip and knee osteoarthritis accounted for 2.4% of all YLD and was ranked as the second most rapidly rising condition associated with disability (31.5% rise in YLD) [2]. The burden is expected to increase due to the aging population and higher rates of obesity. Alcohol consumption is a significant risk factor for the global burden of disease, accounting for 2.2% of age-standardized female deaths and 6.8% of age-standardized male deaths, making it the seventh leading risk factor for both deaths and disability adjusted life years (DALY). 

Based on the Thai Burden of Disease (BOD) Study Group in 2015, the top ten causes of years lived with disability for males were alcohol-use disorders (13.1% of YLD) and osteoarthritis (6.7%), while for females they were osteoarthritis (10.3%) and depression (5.1%). Among females, the total DALY attributable to MDD and osteoarthritis accounted for 1.9% and 3.9%, respectively, while among males, osteoarthritis, and alcohol use disorder (AUD) accounted for 2.0% and 12.0% of total DALY [3]. 

Disability weight is an important parameter used in the calculation of YLD and DALY. It is a measure of the level of disability of a particular health state or disease, with values lying between 0 (full health, no disability) and 1 (disability level in a state such as death) making it a link between mortality and morbidity when estimating DALY [4]. Disability weights are based on subjective rating of disability by respondents’ perception. Overestimation of the disability weight can result in an overestimated DALY while conversely, underestimation can cause an underestimated burden of disease [5]. Differences in social perspectives, treatment methods and changes in disease characteristics over time contribute to different disability weights in different countries. Different valuation methods and sources of respondents can also cause the disability weights to differ for the same disease within the same country [6]. Several methods and instruments to measure health utility values, which can in turn derive disability weights, have been developed and studied, for example the time trade off (TTO) method [7], visual analog scale (VAS) [8], paired comparison method, the European Quality of Life (EQ-5D) [9], and the person trade off (PTO) [10].

Patients with a certain disease of interest can be a good source of information to derive disability weights of that disease as they have experienced the illness both physically and psychologically. Studies have shown that patients’ perceptions of the disability affected by their illness differ between those with mental and physical illnesses [11,12,13]. Patients generally attribute more disability to mental disorders than to physical disorders and the higher disability of mental than physical illness is expressed in terms of social and personal relationships than in functional roles [9]. Furthermore, negative attitudes toward disease history, disability, medication, and negative mood are associated with how people judge their own health [14], which contributes to poor health-related quality of life of patients with either a physical or mental illness [15,16]. Their perceptions of quality of life or disability associated with the illness may be affected by not only the type of illness, its severity and duration, but also by age, sex, and other socio-economic indicators, such as education, employment status, and income, as well as cultural beliefs toward the illness [17,18]. However, there has been little research comparing disability weights calculated from different methods and the effects of socio-demographic and socio-economic status on the disability weights.

The current paper aims to describe and compare the disability weights of the top three diseases (in terms of burden) in Thailand, namely major depressive disorder, alcohol use disorder and osteoarthritis, based on different valuation methods as reported from patients who were currently experiencing those diseases. We selected three valuation methods, namely the visual analogue scale (VAS), the European Quality of Life 5-dimension (EQ-5D) and the time trade-off (TTO) as they allowed the patients to self-report their disability weights. The paired comparison method is used in measurement of disability weights of multiple diseases simultaneously in the general population, while the person-trade-off method required decisions by experts, both of which presented resources and logistical constraints in our study. We decided not to include these methods, focusing instead on the patients’ own perception of their disabilities. In addition, we examined the extent to which type of illness (physical or mental) and socio-economic characteristics affected these disability weights. The results will improve our understanding of valuation methods used to derive disability weights in order to be used in future studies on burden of diseases in Thailand.

## 2. Materials and Methods

### 2.1. Study Design and Participants

We conducted a cross-sectional study on 150 patients diagnosed with mild, moderate, or severe major depressive disorder (ICD-11 code: F32.0, F32.1, or F32.2), 150 patients diagnosed with osteoarthritis of the knee (M17.0) or hip (M16.0), and 150 patients diagnosed with alcohol use disorder (F10.1: harmful use of alcohol, F10.2: alcohol dependence or F10.9: unspecified mental and behavioral disorders, due to the use of alcohol). We recruited patients with MDD and AUD from both a psychiatric hospital and a general hospital and patients with osteoarthritis from a general hospital and a community health center in Songkhla province of southern Thailand between February and July 2018. Osteoarthritis patients were stratified into three groups based on their clinical treatment stated in the medical records; treated with surgery, treated with medication or therapy (physical or occupational), and untreated (waiting for surgery). The AUD patients were interviewed with the Alcohol Use Disorder Identification Test (AUDIT) [19], the results of which were used to classify them into mild (score < 7), moderate (score 8–19), and severe (score >20) level.

Respondents were screened by registered nurses in outpatient clinics. In all disease groups, outpatients aged 18 years or older who visited those hospitals during the study period with those ICD-11 codes and were willing to participate were included. Those who had a tendency toward violent or aggressive behavior and/or severe cognitive impairment were excluded. If respondents met the eligible criteria, they were informed about the study protocol and invited to participate in the study. Patients who agreed to participate signed the consent form before being recruited into the study. We tried to recruit at least 35 participants for each severity level of each disease group. Moreover, we recruited the participants until the total required sample size was reached (n = 150 per disease group). Trained interviewers conducted a face-to-face interview with the patients at the outpatient clinic while they were waiting to meet with the doctor. First, we asked about their personal characteristics (gender, age, education level, income, number of family members) and illness history (duration, experiences of hospitalizations, accidents, and chronic diseases). All patients were then asked to complete the VAS and on completion were asked to complete either the TTO or the EQ-5D instrument, depending on whichever one was randomly assigned to them after recruitment. In each interview, the interviewer first read the lay-person description of the disease state (mild, moderate, or severe) to the patient and asked him/her to confirm his/her current disease state. The patient then completed the VAS and either the TTO or EQ-5D instrument based on that disease state. Each patient completed each instrument only once. The time spent in the interview for each patient was approximately 15 to 35 min.

### 2.2. Valuation Methods

1. In the VAS, patients were asked to rate their current health state on a vertical line, ranging from 0 to 1, with endpoints being: ‘The best health you can imagine’, and ‘The worst health you can imagine’. The disability weight was then obtained from the reversed VAS score as indicated on the vertical line.
2. We used the Thai version of the EQ-5D instrument in this study. Patients were asked to describe their own health state on the five dimensions (questions) which are mobility, self-care, active daily life, pain/discomfort, and anxiety/depression [20]. Each dimension is rated on five levels of difficulty: none, slight, moderate, severe, and extreme. The Thai EQ-5D index scores range from -0.4212 to 1.00, wherein 1 and 0 represent perfect health and death, respectively. Negative values indicate states worse than death [21]. The value obtained from each of the five dimensions forms a single utility score ranging from 0 to 1 and the disability weight is calculated based on a simple linear regression model as follows [21]:

Disability weight = 0.703631 - 0.703631×utility score(1)

3. The TTO method elicits preference scores of a certain health state presented to the respondent by making a trade-off between length of life and quality of life [6]. In this study we limited the disease duration to 10 years using years lived in full health as the anchoring state. We assessed only the health state of better than death, which yielded positive values. The respondent was first asked to compare 10 years living in each severity state of disease to (*x*) years in full health and followed consecutively by a shorter period of time of living in full health until they reached the point of indifference between the two health states: living with disease for another 10 years and living in full health for a shorter period of time. The questions were forced to end each health state with death [22]. An example of a question was “Choice A: stay in a [health state] for 10 years, then die versus Choice B: stay in full health for (x) years (x < 10), then die.” Time (x) was increased by one year until the respondents became indifferent between the two choices. Visual aids for TTO questions were used to help participants reach their point of indifference. The TTO instrument provides a utility score (*u*), which is the division of the time lived with full health (*x*) at the point of indifference by the time lived with that certain disease state (*t*) [23]. Theutility score can then be transformed to a disability weight by subtracting it from 1.

Table 1 illustrates the lay descriptions of all severity levels of three diseases examined in this study. We derived these descriptions from the disability weight study for the global burden of disease in 2013 [4], translated them into Thai, modified some terms in the Thai version to ease understanding by Thai lay-persons, and had the Thai version reviewed by Thai experts in each disease field to assure its comprehensibility among lay-persons. The lay description matched with each respondent’s disease and level of severity was read and explained to him/her before he/she completed the VAS and the other instrument.

### 2.3. Ethics

All subjects gave their informed consent for inclusion before they participated in the study. The study was conducted in accordance with the Declaration of Helsinki, and the protocol was approved by the Ethical Review Committee for Research in Human Subjects, Faculty of Medicine, Prince of Songkhla University [Rec: 60–359–18–1].

### 2.4. Statistical Analysis

Demographic characteristics of the study groups were presented descriptively. The mean disability weights derived from the three methods were compared using an independent samples t-test. We also described disability weights for different levels of severity and compared them using the Kruskal–Wallis one-way analysis of variance and a post-hoc analysis using Dunn’s test. Differences in severity level among the three valuation methods was assessed with Pearson's chi-square test. Lastly, multiple linear regression was used to determine the associations between disability weights and patient’s socio-demographic and illness characteristics after using a directed acyclic graph. Age (in years) was categorized into four groups based on the age distribution of the sample, namely 18–25, 26–40, 41–59, and 60 years and above. Variables having a p-value less than 0.05 were considered as significant. All analyses were performed using R language and environment.

## 3. Results

### 3.1. Patient Characteristics

Table 2 compares demographic characteristics between the three patient groups. MDD patients tended to be younger, had a higher proportion of females, and employed, were more educated, and had a higher monthly family income compared to the other patient groups. Chronic diseases were more common in the osteoarthritis group. Approximately one-third of patients of each disease had been hospitalized or had accidental injuries in last 12 months. Within each disease group, there were no significant differences between the three valuation methods in terms of demographic characteristics, presence of chronic diseases, disease duration, and experiences of hospitalization and accidents.

Among the 150 MDD patients, 48 were diagnosed with mild depressive episode (F32.0), 52 with moderate depressive episode (F32.1), and 50 with severe depressive episode without psychotic symptoms (F32.2). Of the 150 patients with osteoarthritis, 127, and 23 were diagnosed with knee (M17.0) and hip (M16.0) osteoarthritis, respectively. Of the 150 AUD patients, 72 were diagnosed with harmful use of alcohol (F10.1), 60 had alcohol dependence (F10.2), and 18 had other mental and behavioral disorders (F10.9). In addition, 37, 56, and 57 were in the low-risk, moderate-risk and high-risk AUDIT groups, corresponding to mild, moderate, and severe AUD levels, respectively.

Notes: Numbers in table are percentage (IQR) unless stated otherwise. ^†^ P-value < 0.05 for the comparison of demographic characteristics of three patient groups between valuation methods. Abbreviations: IQR, interquartile range; SD, standard deviation; VAS, Visual Analog Scale; EQ-5D, European Quality of Life 5 dimension; TTO, Time Trade-off; MDD, major depressive disorder; AUD, alcohol use disorder.

### 3.2. Disability Weights

#### 3.2.1. Disability Weights by Valuation Method

Table 3 presents the average disability weight of each disease and that by severity level, calculated from the three different valuation methods. Significant differences in the disability weights were seen between three methods for each disease, with VAS giving the highest disability weights and EQ-5D yielding the lowest. Among MDD patients, comparisons of the disability weights between pairs of methods were all significant: VAS vs. TTO (*p* = 0.047), VAS vs. EQ-5D (*p* = 0.001), and TTO vs. EQ-5D (*p* = 0.006). For patients with osteoarthritis, the mean disability weight derived from the VAS was significantly higher than those from the other methods (VAS vs. TTO, *p* < 0.001, VAS vs. EQ-5D, *p* < 0.001); however, there were no significant differences in the disability weights between the TTO and EQ-5D method (*p* = 0.368). Among AUD patients, significant differences were seen with all pairs of methods: VAS vs. TTO (*p* = 0.036), VAS vs. EQ-5D (*p* < 0.001) and TTO vs. EQ-5D (*p* = 0.013).

#### 3.2.2. Disability Weights by Type of Disease

The disability weights for MDD patients ranged from 0.316 to 0.539 and tended to be higher than those for osteoarthritis and AUD by all methods except by the VAS. Based on TTO and EQ-5D methods, osteoarthritis patients had the lowest mean disability weight (0.260 and 0.253, respectively); however, it was highest based on the VAS (0.544). The mean disability weight for AUD patients was in between those for MDD and osteoarthritis patients regardless of valuation method used and ranged between 0.311 and 0.485.

#### 3.2.3. Disability Weights by Severity of Disease

Comparison of disability weights between levels of severity derived from the three methods is shown in Table 3. For all diseases and based on all methods, the disability weights were positively correlated with increasing severity level. Among MDD patients, based on the VAS and EQ-5D methods, those who had severe MDD had a significantly higher mean disability weight than those who had mild MDD (VAS, *p* < 0.001 and EQ-5D, *p* < 0.001, respectively). However, based on the TTO method, there were no significant differences in disability weights between levels of severity of MDD illness (*X*^2^ = 5.435, *p* = 0.06).

Among osteoarthritis patients, based on the EQ-5D and TTO methods, those who were waiting for surgery had a significantly higher mean disability weight than those who had already had surgery (EQ-5D, *p* < 0.001 and TTO, *p* = 0.02) and those treated with medication only (EQ-5D, *p* < 0.001). However, no significant differences in disability weights between levels of disease severity were seen using the VAS (*X*^2^ = 5.566, *p* = 0.06). Similarly, among AUD patients, based on the three methods, those who had severe AUD had a significantly higher mean disability weight than those who had mild AUD (VAS, *p* < 0.001, EQ-5D, *p* < 0.001, and TTO, *p* < 0.001, respectively). 

#### 3.2.4. Association between Respondents’ Characteristics and Disability Weights

Based on the directed acyclic graph there were six variables used for predicting disability weights: age, sex, marital status, disease severity, duration of illness, and history of chronic diseases. Table 4 shows the results of the disease-specific multiple linear regression models examining the relationships between respondents’ characteristics and disability weights obtained from the VAS instrument. Among MDD patients, after adjustment for socio-demographic and illness variables, a significant relationship was seen only for patients with severe disease (estimated = 0.135, p-value < 0.05) compared to those with mild disease. For osteoarthritis patients, no significant relationship was seen for patients’ characteristics and disability weight. Among AUD patients, a significant relationship with disability weight was seen only with the severity level of the disease.

## 4. Discussion

This study examined the disability weights for patients with major depressive disorder, osteoarthritis, and alcohol use disorder, measured by three different valuation methods. Consistent with many previous studies [24,25,26,27], this study found that disability weights calculated using a visual analogue scale were significantly higher than those obtained from other methods, whereas the EQ-5D method resulted in the lowest disability weights [25,27]. The disability weights of mental disorders (MDD and AUD) were also different from that of osteoarthritis, a physical disease, which is consistent with other studies [26]. In contrast to previous studies [14,18], our study found no effect of socio-demographic factors on the disability weights.

Significant differences were seen between disability weights of all three diseases derived from the three valuation methods. We found the same patterns for all three diseases where the TTO method resulted in higher disability weights than the EQ-5D, but lower than the VAS method, which is consistent with other studies [28,29]. This may be explained by differences in the valuation tasks [30], which is a methodological issue and therefore not unexpected [31]. In the VAS, respondents only choose one point on a continuous scale, which reflects their overall perceived health quality on the day of assessment and the disability weight is derived from its reversed score. In contrast, the EQ-5D instrument requires respondents to choose the level of difficulty, based on a categorical scale, for each of the five health dimensions. In both VAS and EQ-5D methods, respondents do not need to trade-off between death, unhealthy life, and/or healthy life in making their decision of the disability score, whereas in the TTO method, respondents have to make a trade-off in order to obtain better health, neither in terms of risk nor in years of life [32]. With these different principles of valuation tasks and in the absence of a gold standard we cannot conclude which method provides the most valid score; thus, systematic assessment of the tools’ validity in the Thai population should be further investigated. However, we can say that all three methods are feasible for determining disability weights among Thai patients, which was one of the aims of our study. In addition, in all three diseases we found positive correlations between illness severity and disability weight derived from each of the three methods. This could, in a way, support the high face validity of the disability weights estimated in our study [33].

As seen in other studies [11,12,13], patients with mental diseases, especially major depression, had higher disability weights than those with osteoarthritis, a physical disease. The perception of patients affects their judgement of their disability associated with an illness. The results of the most recent World Mental Health surveys found that patients with mental disorders generally contributed more disability to their mental illnesses than to their physical illnesses [11]. It was also shown that the reduction of perceived health is similar for physical and mental conditions, but the pattern of mediation is different. For example, stigma and family size are significantly associated with mental illnesses, while mobility is significantly associated with physical illnesses [34]. In addition, mental illnesses can cause higher disability than physical illnesses because the former are more related with social and interpersonal relationships than with functional roles [11]. Some physical illnesses, such as diabetes and osteoarthritis, generally do not affect patient’s thinking, behavior, or perception of the illness, whereas mental illnesses, such as depression, affect the individual’s self-perception and self-image through their illness experiences and severity [35].

Patient’s characteristics might affect how people estimate their health condition or disability weights [36]. In contrast with other studies, our study did not find any socio-demographic or economic characteristics associated with disability weights. This may imply that no socio-demographic characteristics have an impact on patients’ perceptions of their disability but it depends on the severity of their illness. However, the sample size of our study was rather small, the lack of significant associations might be because of inadequate power.

There are a few limitations in this study. First, this study was exploratory and hospital-based. Therefore, generalizability may be limited and results need to be interpreted with caution. Second, TTO elicitation has a high cognitive burden for patients; therefore, interviewer bias, socially desirability bias, and acquiescence bias may have occurred, especially for mental health patients [37]. For example, when we asked patients to trade length of life with quality of life, some were unable to answer and stopped the interview. Another limitation is our use of a lay description of health states, which were used for confirming patients’ current symptoms. Understanding of the description of a disease-specific state may be difficult for some patients because of the potential variation in the severity of the disease and the disease’s impact on a patient’s life [33]. The disability weights may thus depend on how much respondents understand the disease description and perceive their current health status [37].

Future studies should consider systematic assessment of the tools’ validity in the Thai population. The technique of applying valuation methods also needs to be applied in large population groups to keep random measurement error at an acceptable level and can be implied for the whole population.

## 5. Conclusions

Patients’ perceptions of their disability differ between diseases, disease severity, and by valuation method. Either disease conditions or valuation methods may simply reflect the heterogeneity in people’s preferences regarding their own health state. Patients’ socio-demographic and economic characteristics may have no effect on their perceptions of their disability or health condition. Our findings highlight the importance of methods for elicitation of disability weights, which can rely on disease specific conditions (mental illness versus physical illness). More studies are needed to investigate whether there are variations between disability weights for different types of illnesses, patients’ perceptions of their disability, and patient-and disease-specific factors.

## Figures and Tables

**Table 1:** Lay descriptions for all severity levels of three diseases; namely alcohol use disorder, major depressive disorder, and osteoarthritis, examined in the study.

Health State	Lay Description
Alcohol-use disorder severity	
Mild	Drinks a lot of alcohol and sometimes has difficulty controlling the urge to drink. While intoxicated, the person has difficulty performing daily activities.
Moderate	Drinks a lot, gets drunk almost every week and has great difficulty controlling the urge to drink. Drinking and recovering causes great difficulty in daily activities, and causes sleep loss and fatigue.
Severe	Gets drunk almost every day and is unable to control the urge to drink. Drinking and recovering replace most daily activities. The person has difficulty thinking, remembering, and communicating, and feels constant pain and fatigue.
Major depressive disorder severity	
Mild	Feels persistent sadness and has lost interest in usual activities. The person sometimes sleeps badly, feels tired, or has trouble concentrating but still manages to function in daily life with extra effort.
Moderate	Has constant sadness and has lost interest in usual activities. The person has some difficulty in daily life, sleeps badly, has trouble concentrating, and sometimes thinks about harming himself (or herself).
Severe	Has overwhelming, constant sadness and cannot function in daily life. The person sometimes loses touch with reality and wants to harm or kill him/herself.
Osteoarthritis category	
Treated with surgery	The person has already had surgery for total knee/hip replacement and supportive treatment (pain medication, anti-inflammatories), which resulted in reduced pain and disability.
Treated with medication/therapy	Has pain in knee or hip, causing difficulty to move the knee or walk with a limp and feels discomfort when walking. Supportive treatment (pain medication, anti-inflammatories) may result in reduced pain and disability.
No treatment (waiting for surgery	Has severe pain in knee or hip, causing difficulty to move the knee or walk with a limp and feels discomfort when walking. Supportive treatment (pain medication, anti-inflammatories) may result in reduced pain and disability. Joint replacement may occur and patients are waiting for surgery of total knee/hip replacement.

**Table 2:** Comparison of demographic characteristics of three patient groups by valuation method.

Characteristic	MDD Patients (*n* = 150)				Osteoarthritis Patients (*n* = 150)				AUD Patients (*n* = 150)			
	EQ–5D (*n* = 75)	TTO (*n* = 75)	VAS (*n* = 150)	*p*– value	EQ–5D (*n* = 75)	TTO (*n* = 75)	VAS (*n* = 150)	*p*– value	EQ–5D (*n* = 75)	TTO (*n* = 75)	VAS (*n* = 150)	*p*– value
Sex												
Male	38.7 (27.5,49.8)	30.7 (20.1,41.2)	34.7 (27.0,42.3)	0.303	17.4 (8.3,26.4)	22.1 (12.1,32.0)	19.7 (13.0,26.4)	0.492	84.0 (75.6, 92.4)	78.7 (69.3, 88.0)	81.3 (75.1, 87.6)	0.402
Female	61.3 (50.2,72.5)	69.3 (58.8,79.9)	65.3 (57.7,73.0)		82.6 (73.6,91.7)	77.9 (68.0,87.9)	80.3 (73.6,87.0)		16.0 (7.6, 24.4)	21.3 (12.0, 30.7)	18.7 (12.4, 24.9)	
Age (years) mean (sd)	51.2 (16.2)	47.9 (17.8)	49.5 (17.1)	0.243	64.8 (11.8)	63.1 (11.4)	64.0 (11.6)	0.631	50.8 (14.6)	53.6 (16.4)	52.2 (15.5)	0.266
Education level												
No formal education	2.7 (1.0,6.4)	1.3 (1.2,4.0)	2.0 (0.3,4.3)	0.476	4.3 (1.0,9.2)	2.9 (1.1,7.0)	3.6 (0.5,6.8))	0.333	2.8 (1.0,6.4)	2.7 (1.0, 6.4)	2.7 (0.0,5.0)	0.510
Primary school	49.3 (37.9,60.8)	41.3 (30.1,52.6)	45.3 (37.3,53.4)		50.7 (38.8,62.7))	63.2 (51.6,74.8)	56.9 (48.6,65.3)		41.3 (30.1,52.6)	50.7 (39.2,62.1)	46.0 (38.0,54.0)	
Secondary school or higher	48.0 (36.6,59.4)	57.3 (46.0,68.6)	52.7 (44.6,60.7)		44.9 (33.1,56.8)	33.8 (22.4,45.2)	39.4 (31.2,47.7)		56.0 (44.6,67.4)	46.7 (35.3,58.1)	51.3 (30.8,46.5)	
Marital status												
Married	58.7 (47.4,69.9)	61.3 (50.2,72.5)	60.0 (52.1,67.9)	0.739	72.5 (61.8,83.1)	61.8 (50.1,73.4)	67.2 (59.2,75.1)	0.182	74.7 (64.7, 84.6)	65.3 (54.4,76.2)	70.0 (62.6,77.4)	0.212
Single	41.3 (30.1,52.6)	38.7 (27.5,49.8)	40.0 (32.1,47.9)		27.5 (16.9,38.2)	38.2 26.6,49.9)	32.8 (24.9,40.8)		25.3 (15.4,35.3)	34.7 (23.8,45.6)	30.0 (22.6, 37.4)	
Occupation												
Unemployed	46.7 (35.3,58.1)	46.8 (34.3,59.0)	46.7 (38.6,54.7)	0.814	36.2 (24.8,47.7)	47.1 (35.1,59.1)	41.6 (33.3,49.9)	0.199	20.0 (10.8, 29.2)	29.3 (18.9, 39.7)	24.7 (26.4, 41.6)	0.185
Employed	53.3 (41.9,64.7)	53.2 (42.9,64.4)	53.3 (45.3,61.4)		63.8 (52.3,75.2)	52.9 (40.9,64.9)	58.4 (50.1,66.7)		80.0 (70.8, 89.2)	70.7 (60.3, 81.1)	75.3 (68.4, 82.3)	
Income (baht)												
median	9000	7000	8000	0.467	9000	6500	8000	0.364	7000.0	5000.0	6000.0	0.321
IQR	3000–18,000	2000– 20,000	2700–18,000		3000–17,000	1750–18,500	2000–17,000		3000–18,000	800–12,000	2000–15,000	
Family size (mean, SD)	3.3 (1.6)	3.3 (1.4)	3.3 (1.5)	0.773	3.3 (2.1)	3.4 (1.5)	3.4 (1.5)	0.863	3.8 (1.6)	3.4 (2.0)	3.6 (1.8)	0.210
Chronic diseases	53.3 (41.9,64.7)	48.0 (36.6,59.4)	50.7 (42.6,58.7)	0.514	75.4 (65.1,85.6)	67.6 (56.4,789)	71.5 (63.9,79.1)	0.317	36.0 (25.0, 47.0)	38.7 (27.5, 49.8)	37.3 (29.5, 45.1)	0.736
Duration of illness (years); mean (SD)	0.6(0.3)	0.7 (0.3)	0.6(0.3)	0.149	2.4(1.5)	2.6(1.6)	2.5(1.5)	0.328	1.0(0.5)	1.1(0.5)	1.1(0.5)	0.139
Hospitalization	37.3 (26.3,48.4)	36.0 (25.0,47.0)	36.7 (28.9,44.4)	0.865	34.8 (23.4,46.1)	36.8 (25.2,48.4)	35.8 (27.7,43.9)	0.809	30.7 (20.1,41.2)	32.0 (21.3,42.7)	31.3 (23.9,38.8)	0.860
Accident	32.0 (21.3,42.7)	33.3 (22.5,44.1)	32.7 (25.1,40.2)	0.862	29.0 (18.2,39.8)	22.1 (12.1,32.0)	25.5 (18.2,32.9)	0.353	36.0 (25.0,47.0)	30.7 (20.1,41.2)	33.3 (25.7,40.9)	0.488

**Table 3:** Disability weights of patients based on visual analog scale, EQ5D, and time-trade off methods by severity level of diseases.

	MDD Patients				Osteoarthritis Patients				AUD Patients			
Valuation method	Mild	Moderate	Severe	Total	Treated with Surgery	Treated with Medication	Waiting for Surgery	Total	Mild	Moderate	Severe	Total
VAS	0.482 ^a^ (0.229)	0.515 ^b^ (0.226)	0.617 ^ab^ (0.204)	0.539 (0.226) ^d†^	0.486 (0.288)	0.541 (0.245)	0.645 (0.238)	0.544 (0.262) ^d†^	0.395 ^ab^ (0.213)	0.511 ^a^ (0.153)	0.546 ^b^ (0.282)	0.485 (0.231) ^d†^
EQ-5D	0.212 ^a^ (0.199)	0.235 ^b^ (0.177)	0.500 ^ab^ (0.253)	0.465 (0.406) ^e†^	0.120 ^a^ (0.093)	0.185 ^b^ (0.135)	0.484 ^ab^ (0.280)	0.260 (0.302) ^e^	0.212 ^a^ (0.256)	0.252 ^b^ (0.241)	0.450 ^ab^ (0.262)	0.405 (0.330) ^e†^
TTO	0.342 (0.356)	0.512 (0.433)	0.536 (0.410)	0.316 (0.247) ^f†^	0.246 ^ab^ (0.353)	0.256 ^a^ (0.241)	0.280 ^b^ (0.316)	0.253 (0.238) ^f†^	0.318 ^a^ (0.386)	0.398 (0.319)	0.498 ^a^ (0.261)	0.311 (0.271) ^f†^

Notes: Superscripts (a, b and c) appearing in the table indicate significant pairwise differences between disease severity groups (within valuation method) based on Dunn’s test. ^d^ VAS vs TTO, ^e^ TTO vs EQ-5D, ^f^ VAS vs EQ-5D. ^†^ *p*-value < 0.05 for the comparison of mean disability weights between valuation methods (paired t-test). Abbreviations: SD, standard deviation; VAS, Visual Analog Scale; EQ-5D, European Quality of Life 5 dimension; TTO, Time trade-off; MDD, major depressive disorder; AUD, alcohol use disorder.

**Table 4:** Multivariate linear regression model predicting disability weights based on the VAS method.

Characteristic	MDD		Osteoarthritis		AUD	
	Estimate	*p*-value	>Estimate	*p*-value	>Estimate	*p*-value
	(95% CI)		(95% CI)		(95% CI)	
Age group (years)^†^						
26–40	−0.053 (0.188, 0.081)	0.433	−0.467 (−1.114,0.181)	0.157	−0.164 (−0.488,0.159)	0.317
41–59	−0.005 (−0.140, −.130)	0.938	−0.051 (−0.440,0.338)	0.797	−0.055 (−0.377,0.266)	0.733
≥60	−0.081 (−0.211,0.049)	0.222	−0.080 (−0.458,0.299)	0.677	−0.146 (−0.469,0.177)	0.373
Sex (Female) ^†^	−0.0159 (−0.092,0.060)	0.680	0.027 (−0.084,0.140)	0.629	0.019 (−0.082,0.119)	0.714
Marital Status (Married) ^†^	−0.021 (−0.105,0.062)	0.617	−0.066 (−0.162,0.031)	0.181	0.014 (−0.069,0.096)	0.745
Severity level						
Mode rate	0.038 (−0.60,0.136)	0.448	0.021 (−0.009,0.132)	0.705	0.110 (0.017,0.202)	0.021
Severe	0.135 (0.041,0.229)	0.005	0.099 (−0.014,0.212)	0.086	0.145 0.050,0.241)	0.003
Duration of illness (years)	−0.003 (−0.013,−0.007)	0.553	0.002 (−0.001,0.004)	0.168	0.003 (−0.010,0.001)	0.177
Chronic disease(s) (yes/no)	−0.066 (−0.142,0.010)	0.088	0.062 (−0.034,0.160)	0.203	0.020 (−0.064,0.099)	0.673

Notes: ^†^ Reference groups for categorical variables are as follows: Age group (18–25), Sex (Male), Marital status: (single), Severity level: (mild). Abbreviations: SD, standard deviation; VAS, Visual Analog Scale; EQ-5D, European Quality of Life 5 dimension; TTO, Time Trade-off; CI, confidence interval; MDD, major depressive disorder; AUD, alcohol use disorder.
